# Health Assessment Questionnaire-Disability Index (HAQ-DI) use in modelling disease progression in diffuse cutaneous systemic sclerosis: an analysis from the EUSTAR database

**DOI:** 10.1186/s13075-020-02329-2

**Published:** 2020-10-28

**Authors:** Yannick Allanore, Sylvie Bozzi, Augustin Terlinden, Doerte Huscher, Caroline Amand, Christina Soubrane, Elise Siegert, László Czirják, Patricia E. Carreira, Eric Hachulla, Elisabetta Zanatta, Mengtao Li, Paolo Airò, Fabian A. Mendoza, Edoardo Rosato, Oliver Distler, on behalf of the EUSTAR Collaborators, Svetlana Agachi, Walid Ahmed Abdel Atty Mohamed, Paolo Airò, Sabine Adler, Juan Jose Alegre-Sancho, Yannick Allanore, Lidia P. Ananieva, Codrina Ancuta, Branimir Anic, Fabiola Atzeni, Gianluigi Bajocchi, Alexandra Balbir-Gurman, Marko Baresic, Radim Becvar, Laura Belloli, Vera Bernardino, Washington Bianchi, Breno Valdetaro Bianchi, Otylia Kowal Bielecka, Francesco Paolo Cantatore, Patricia E. Carreira, Ivan Castellví, Marco Matucci Cerinic, Carlo Chizzolini, Lorinda S. Chung, Bernard Coleiro, Maura Couto, Mary Ellen Csuka, Giovanna Cuomo, Maurizio Cutolo, László Czirják, Lorenzo Dagna, Nemanja Damjanov, Ellen De Langhe, Francesco Del Galdo, Carlos de la Puente, Christopher Denton, Carolina de Souza Müller, Jeska de Vries-Bouwstra, Jörg Distler, Oliver Distler, Andrea Doria, Merete Engelhart, Kilian Eyerich, Nihal Fathi, Dominique Farge Bancel, Rosario Foti, Daniel Furst, Armando Gabrielli, Francis Gaches, Paloma García de la Peña Lefebvre, Ana Maria Gheorghiu, Alessandro Giollo, Brigitte Granel, Eric Hachulla, Paul Hasler, Stefan Heitmann, Jörg Henes, Ariane Herrick, Kristine Herrmann, Roger Hesselstrand, Anna-Maria Hoffmann-Vold, Ivien M. Hsu, Nicolas Hunzelmann, Florenzo Iannone, Francesca Ingegnoli, Massimiliano Limonta, Ruxandra Maria Ionescu, Murat Inanc, Patrick Jego, Sergio Jimenez, Jorge Juan Gonzalez Martin, Sarah Kahl, Cristiane Kayser, Eduardo Kerzberg, Michaela Kohm, Ina Kötter, Dorota Krasowska, Brigitte Krummel-Lorenz, Eugene J. Kucharz, Yair Levy, Mengtao Li, Ira Litinsky, Esthela Loyo, Dominik Majewski, Thierry Martin, Julia Martínez-Barrio, Mathieu D’Alessandro, Miroslav Mayer, Arsene Mekinian, Fabian Mendoza, Sergey Moiseev, Andrea Lo Monaco, C. Montecucco, Jadranka Morovic-Vergles, Luc Mouthon, Ulf Müller-Ladner, Srdan Novak, Pavel Novikov, Fahrettin Oksel, Marzena Olesinska, Susana Oliveira, Kati Otsa, Omer Nuri Pamuk, Raffaele Pellerito, Katja Perdan-Pirkmajer, Carmen Pizzorni, Hadi Poormoghim, Maria Rosa Pozzi, Francesco Puppo, Piercarlo Sarzi Puttini, Mislav Radic, Ana-Maria Ramazan, Simona Rednic, Elena Rezus, Valeria Riccieri, Gabriela Riemekasten, Doron Rimar, Edoardo Rosato, Maria João Salvador, Nora Sandorfi, Vera Ortiz Santamaria, Petra Saar, Lesley Ann Saketkoo, Percival D. Sampaio-Barros, Tim Schmeiser, Raffaella Scorza, Matthias Seidel, Carlo Francesco Selmi, Goda Seskute, Petros Sfikakis, Ami Sha, Jean Sibilia, Elise Siegert, Vanessa Smith, Kamal Solanki, François Spertini, Bojana Stamenkovic, Lisa Stamp, Simon Stebbings, Jiri Stork, Cord Sunderkötter, Gabriela Szücs, Cristina-Mihaela Tanaseanu, Mohammed Tikly, Carmen Tineo, Marie-Elise Truchetet, Susanne Ullman, Maria Üprus, Alessandra Vacca, Jacob van Laar, Marie Vanthuyne, Gabriele Valentini, J. M. van Laar, Douglas Veale, Peter Villiger, P. Vlachoyiannopoulos, Carlos Alberto von Mühlen, Ulrich Walker, Piotr Wiland, Figen Yargucu, Sule Yavuz, Elisabetta Zanatta, Zbigniew Zdrojewski, Thierry Zenone

**Affiliations:** 1 Service de Rhumatologie, Cochin Hospital, APHP, Paris Descartes University, 27 rue du Faubourg Saint Jacques, 75014 Paris, France; 2 Sanofi R&D, Chilly-Mazarin, France; 3 Institute of Biometry and Clinical Epidemiology and Berlin Institute of Health, Charité - Universitaetsmedizin, Berlin, Germany; 4 Charité University Hospital, Berlin, Germany; 5 University of Pécs, Medical School, Pécs, Hungary; 6 Hospital University 12 October, Madrid, Spain; 7 University of Lille, Claude Huriez’ Hospital, Lille Cedex, France; 8 University of Padova, Padova, Italy; 9 Peking Union Medical College Hospital, Chinese Academy of Medical Sciences, Beijing, P.R. China; 10 Rheumatology and Clinical Immunology, Spedali Civili, Brescia, Italy; 11 Jefferson Institute of Molecular Medicine, Thomas Jefferson University, Philadelphia, PA USA; 12 La Sapienza University, Polyclinic Umberto I, Rome, Italy; 13 Department of Rheumatology, University Hospital Zurich, Zurich, Switzerland

**Keywords:** EUSTAR registry, Diffuse cutaneous systemic sclerosis, Health Assessment Questionnaire-Disability Index, HAQ-DI score

## Abstract

**Background:**

Patients with diffuse cutaneous systemic sclerosis (dcSSc) have a poor prognosis. The importance of monitoring subjective measures of functioning and disability, such as the Health Assessment Questionnaire-Disability Index (HAQ-DI), is important as dcSSc is rated by patients as worse than diabetes or hemodialysis for quality of life impairment. This European Scleroderma Trials and Research (EUSTAR) database analysis was undertaken to examine the importance of impaired functionality in dcSSc prognosis. The primary objectives were to identify predictors of death and HAQ-DI score progression over 1 year. HAQ-DI score, major advanced organ involvement, and death rate were also used to develop a comprehensive model to predict lifetime dcSSc progression.

**Methods:**

This was an observational, longitudinal study in patients with dcSSc registered in EUSTAR. Death and HAQ-DI scores were, respectively, analyzed by Cox regression and linear regression analyses in relation to baseline covariates. A microsimulation Markov model was developed to estimate/predict natural progression of dcSSc over a patient’s lifetime.

**Results:**

The analysis included dcSSc patients with (*N* = 690) and without (*N* = 4132) HAQ-DI score assessments from the EUSTAR database. Baseline HAQ-DI score, corticosteroid treatment, and major advanced organ involvement were predictive of death on multivariable analysis; a 1-point increase in baseline HAQ-DI score multiplied the risk of death by 2.7 (*p* <  0.001) and multiple advanced major organ involvement multiplied the risk of death by 2.8 (*p* <  0.05). Multivariable analysis showed that baseline modified Rodnan Skin Score (mRSS) and baseline HAQ-DI score were associated with HAQ-DI score progression at 1 year (*p* <  0.05), but there was no association between baseline organ involvement and HAQ-DI score progression at 1 year. HAQ-DI score, major advanced organ involvement, and death were successfully used to model long-term disease progression in dcSSc.

**Conclusions:**

HAQ-DI score and major advanced organ involvement were comparable predictors of mortality risk in dcSSc. Baseline mRSS and baseline HAQ-DI score were predictive of HAQ-DI score progression at 1 year, indicating a correlation between these endpoints in monitoring disease progression. It is hoped that this EUSTAR analysis may change physician perception about the importance of the HAQ-DI score in dcSSc.

## Introduction

Systemic sclerosis (SSc) is an autoimmune disorder characterized by extensive fibrosis and vasculopathy that can affect the skin and internal organs [1]. It imposes a substantial burden of pain, disfigurement, and impaired functionality that can markedly reduce health-related quality of life [2, 3]. SSc is a heterogeneous disease, and it is commonly subclassified according to the extent of skin involvement [4]. Patients with diffuse cutaneous systemic sclerosis (dcSSc) have the poorest prognosis, with a 10-year mortality rate of at least 50% [5]. It is, therefore, important to monitor these patients in order to provide a comprehensive and long-term assessment of disease progression.

To date, the modified Rodnan Skin Score (mRSS) has been widely used to monitor progression in clinical studies; it is a semi-quantitative measure of skin thickness in different body areas that is used as a proxy for disease severity, progression, and mortality risk [6, 7]. Short-term progressive skin fibrosis (i.e., within 1 year measured via mRSS) is associated with a later decline in lung function and worse survival in patients with dcSSc [7]. However, the progression of skin fibrosis diverges from other organ involvement during the disease course. It often regresses spontaneously once it has reached its peak, while lung fibrosis continues to progress in most cases. This can make short-term clinical trials of therapeutic agents challenging as outcomes reported with the mRSS do not always correlate with other objective measures [8, 9].

Ensuring that the patient voice is heard is also central to decision making. Patients with SSc have strong views about the chronic nature and negative consequences of their disease. They perceive the impact of their disease on daily living as more severe than patients with diabetes or those undergoing hemodialysis [10]. However, patient opinion is often not satisfactorily included in decision making [2]. Patient-reported outcomes have become increasingly important in recent years; they could address this imbalance and increase our understanding of the wider impact of these diseases.

Accumulating evidence suggests that the Health Assessment Questionnaire-Disability Index (HAQ-DI) is a useful measure of functionality in SSc [11–13]. This is a self-reported questionnaire covering 20 items in eight domains related to measuring difficulty in performing activities of daily living: dressing, arising, eating, walking, hygiene, reach, grip, and common daily activities [12, 13]. Each question is rated on a 0–3 scale, where 0 indicates “without difficulty” and 3 indicates “unable to do,” and additional points can be added if aids or devices are needed for specific activities; thus, increasing score indicates worse functionality [12, 13]. HAQ-DI score is a reliable measure that is sensitive to change in disease activity in cross-sectional (patients and physicians) and longitudinal studies [2, 12]. As a result, it is now increasingly used as an endpoint in clinical studies investigating treatment outcomes in SSc.

The primary aims of this European Scleroderma Trials and Research (EUSTAR) database analysis of patients with dcSSc were (1) to identify predictors of death including HAQ-DI score, advanced major organ involvement, and other clinical characteristics; (2) to identify predictors of HAQ-DI score progression over 1 year; and (3) to develop a transition model to predict natural progression of dcSSc over a lifetime using HAQ-DI score, major advanced organ involvement, and death rates.

## Methods

### Design and study population

This was an observational, longitudinal study of patients with dcSSc registered in the EUSTAR database. The EUSTAR network has been described elsewhere [14, 15]. In brief, EUSTAR is a growing database of patients with scleroderma treated at centers worldwide; all patients undergo annual scheduled clinic visits, providing observational, longitudinal data.

For this analysis, patient data were extracted from January 1995 to February 2019. Ethics Committee approval was obtained from all centers, and informed consent was provided when required by the ethical regulations at the specific centers. Patients were ≥ 18 years of age and to have SSc as classified by the American College of Rheumatology (ACR) (1980) or ACR/European League Against Rheumatism criteria (2013) [16, 17]. Patients with dcSSc were identified from this cohort based on the LeRoy criteria [4], and those with the available data for the first non-Raynaud’s manifestation were extracted. If data on the LeRoy criteria were not available, then the extent of skin involvement (e.g., skin fibrosis at any time with mRSS ≥ 1 of upper arms, thorax, abdomen, or thighs) was used as a surrogate.

### Population and outcomes

**Fig. 1:**
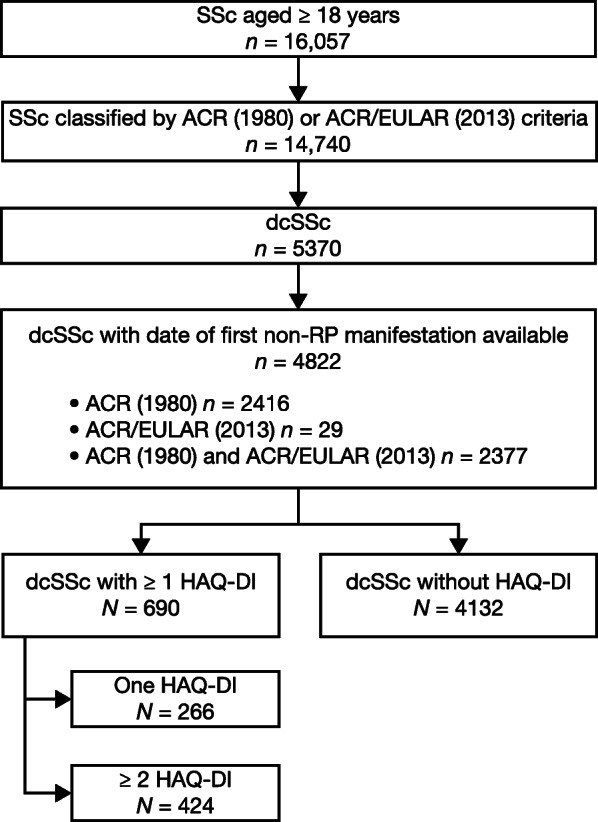
EUSTAR cohort with dcSSc (February 11, 2019). *ACR* American College of Rheumatology, *ACR/EULAR* American College of Rheumatology/European League Against Rheumatism, *dcSSc* diffuse cutaneous systemic sclerosis, *EUSTAR* European Scleroderma Trials and Research, *HAQ-DI* Health Assessment Questionnaire-Disability Index, *RP* Raynaud’s phenomenon, *SSc* systemic sclerosis

Analyses were performed using patients with dcSSc who met the above criteria with or without HAQ-DI score assessments. Patients with HAQ-DI score were further divided into either those who had ≥ 1 HAQ-DI or those who had ≥ 2 HAQ-DI score assessments. Clinical data used in analyses are shown in Table 1, Fig. 1, and Fig. S1.

**Table 1:** Baseline demographic and clinical characteristics according to HAQ-DI score assessments

Parameters	dcSSc with no HAQ-DI score at any time *N* = 4132	dcSSc with ≥ 1 HAQ-DI score *N* = 690	dcSSc with ≥ 2 HAQ-DI scores *N* = 424
Age (years)			
Mean (SD)	52.2 (13.6)	53.8 (12.9)	53.1 (12.7)
IQR	42.6–62.4	45.6–62.6	43.9–62.3
Age (years) at onset of RP, *N*	3953	677	414
Mean (SD)	43.3 (14.7)	42.4 (14.4)	42.5 (14.3)
IQR	32.7–54.0	32.4–52.0	32.1–52.3
Female, *n* (%)	3214 (77.8%)	537 (77.8%)	320 (75.5%)
Disease duration, *n* (%)			
≤ 18 months	962 (23.3%)	60 (8.7%)	39 (9.2%)
> 18 months	3170 (76.7%)	630 (91.3%)	385 (90.8%)
Current digital ulcers, *n* (%)			
Yes	1493 (36.6%)	120 (18.3%)	70 (17.3%)
No	2585 (63.4%)	535 (81.7%)	335 (82.7%)
Puffy fingers, *n* (%)			
Yes	972 (48.7%)	216 (35.5%)	148 (39.1%)
No	1024 (51.3%)	392 (64.5%)	231 (60.9%)
mRSS, *N*	3895	656	403
Mean (SD)	16.4 (9.9)	11.7 (9.0)	11.8 (8.6)
IQR	9.0–23.0	5.0–17.0	5.0–17.0
HAQ-DI score			
Mean (SD)	NA	1.0 (0.8)	1.1 (0.8)
IQR	NA	0.4–1.6	0.4–1.8
Advanced organ involvement, *n* (%)			
None	3448 (83.7%)	468 (68.8%)	295 (69.9%)
One	585 (14.2%)	178 (26.2%)	108 (25.6%)
Multiple	86 (2.1%)	34 (5.0%)	19 (4.5%)
Missing	13	10	2

*dcSSc* diffuse cutaneous systemic sclerosis, *HAQ-DI* Health Assessment Questionnaire-Disability Index, *IQR* interquartile range, *mRSS* Modified Rodnan Skin Score, *NA* not available, *RP* Raynaud’s phenomenon, *SD* standard deviation

Analyses of the impact of organ involvement focused on the major advanced complications seen in dcSSc patients. Advanced gastrointestinal (GI) events were defined as malabsorption or ≥ 10% weight loss from baseline. Echocardiographic measurement of systolic pulmonary arterial pressure > 45 mmHg was used as a proxy for pulmonary hypertension (PH) as data on right heart catheterization were not collected routinely in the database. Interstitial lung disease (ILD) was confirmed by high-resolution computed tomography or chest X-ray, and significant lung involvement was defined as ILD on imaging and forced vital capacity < 75% predicted. Cardiac involvement was defined as left ventricular ejection fraction (LVEF) ≤ 45% measured on echocardiography, and renal involvement was defined as the presence of renal crisis (abrupt onset of severe hypertension accompanied by rapidly progressive renal failure, hypertensive encephalopathy, congestive heart failure, and/or microangiopathic hemolytic anemia)*.*

Patients could be enrolled at any time in the disease course if they were classified as having SSc (defined as baseline). Clinical history, demographic characteristics, use of immunomodulatory treatment, and death were documented for patients with or without HAQ-DI score assessments; this information was based on medical records in the EUSTAR database. It was intended that patients should be documented once a year. Death was documented with the date or year (if the date was not exactly known) and reason of death.

### Statistical analyses

#### Regression analyses to identify predictors of death and HAQ-DI score progression

Patients with ≥ 2 HAQ-DI score assessments were selected to model prediction of HAQ-DI score at 1 year (i.e., progression). Patients with ≥ 2 specific organ assessments were selected to predict major advanced organ involvement. Using these populations, the relationship between baseline characteristics and death rates and HAQ-DI score progression at 1 year were examined both at the univariate level and in multivariable models. Death rates and HAQ-DI scores were analyzed by Cox regression and linear regression analyses, respectively, in relation to each baseline covariate: gender, age at onset of Raynaud’s phenomenon, RNA polymerase III positive, immunomodulator treatment, corticosteroid use > 10 mg/day, HAQ-DI score, mRSS, and number of major advanced organs involved (0, 1, or ≥ 2 based on lung, cardiac, GI, PH, or renal involvement). All analyses were performed using IBM® SPSS statistics 24.0 and R software (version 3.4.4).

#### Transition model for dcSSc

A microsimulation Markov model was developed to determine the natural progression of dcSSc over a patient’s lifetime horizon. The Markov model was chosen as it enables simulation of a patient in different health states (e.g., various HAQ-DI levels, organ involvement or not, and alive or dead). Each patient was modelled independently to allow for the heterogeneity of the dcSSc population; it was, therefore, a microsimulation. Within the abovementioned dataset, three risk equations were developed to model the evolution of the following endpoints: HAQ-DI score; major advanced organ involvement; death.

The HAQ-DI score is a continuous variable and was transformed into a categoric variable in this model to represent five different health states ([0− 0.5], [0.5− 1.0], [1.0− 1.5], [1.5− 2.0], and [2.0− 3.0]); the health states were based on expert opinion and were initially defined as uniformly splitting the 0− 3 HAQ-DI score range into six categories (i.e., this is the maximum amount of categories that enable the multistate modelling [MSM] package to converge when fitting a transition matrix between HAQ-DI states); however, the two last categories did not contain enough patients and were grouped into a single state, leaving five states in total. The HAQ-DI transition matrix was calibrated using an MSM that provides constant transition probabilities over time from any HAQ-DI state to any other one. For calibration of the HAQ-DI transition matrix, transition intensities were developed using longitudinal, patient-level data for HAQ-DI states as a function of gender, age at baseline, and lung status at baseline. A 1-year cycle was chosen to transform intensities into yearly transition probabilities of moving between the five HAQ-DI states (Table S1).

For calibration of the organ equations, longitudinal patient-level data were used to calculate survival in the no-organ-involved state in relation to specific patient characteristics at baseline (age, sex, HAQ-DI states, and major advanced organ involvement such as lung, PH, cardiac, renal, or GI) (Table S2). This equation was calibrated using the survival package (R software).

**Fig. 2:**
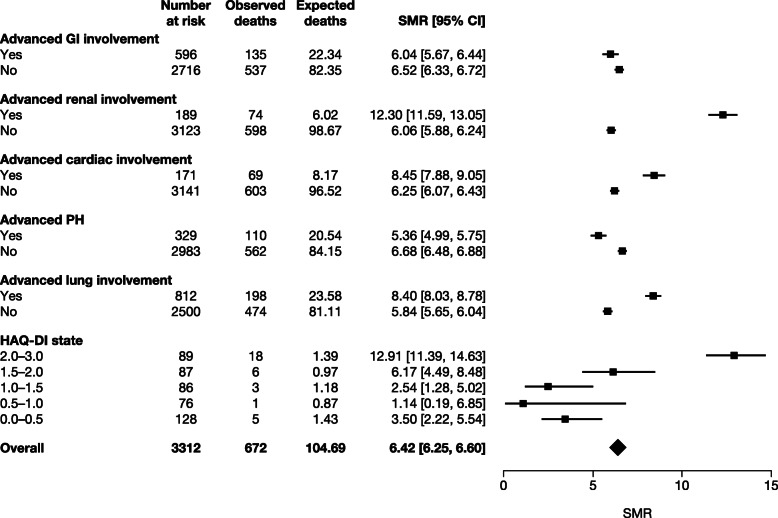
Calculation of SMR using death rates from the EUSTAR dcSSc cohort (observed deaths) and deaths in the general population (expected deaths from the Italian life tables) in patients with ≥ 1 HAQ-DI score measurement. *CI* confidence interval, *dcSSc* diffuse cutaneous systemic sclerosis, *EUSTAR* European Scleroderma Trials and Research, *GI* gastrointestinal, *HAQ-DI* Health Assessment Questionnaire-Disability Index, *PH* pulmonary arterial hypertension, *SMR* standardized mortality ratio

For calibration of the third equation, death rates were calculated using a standardized mortality ratio (SMR), defined as the ratio of observed deaths in the dcSSc EUSTAR population to expected deaths in the general population (adjusted for age- and gender-specific rates) (Fig. 2). Thus, SMRs differ from hazard ratios as these compare the mortality of specific dcSSc patients with the mortality of other dcSSc patients. The general population mortality data were extracted from the 2014 Italian life tables [18] as Italian was the most prevalent nationality (22%) in the analyzed cohort from the EUSTAR database at the time of analysis [19]. This was used as a mortality multiplier, sensitive to the fact that a patient is in a specific HAQ-DI state or any advanced organ state.

**Fig. 3:**
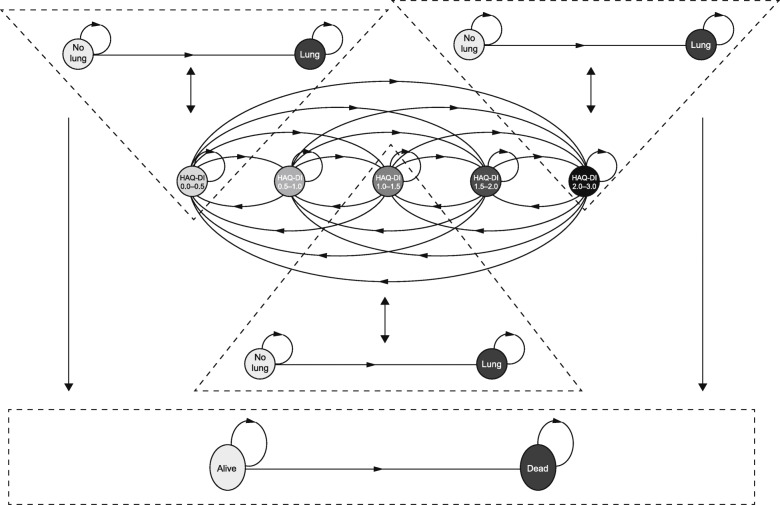
Illustration of HAQ-DI transition states (0–0.5, 0.5–1.0, 1.0–1.5, 1.5–2.0, and 2.0–3.0), risk of advanced lung involvement, and death for a patient with dcSSc. HAQ-DI state in *t* − 1 influences the lung status in *t*. Similarly, the lung status in *t* − 1 influences the HAQ-DI state in *t*. The lung status in *t* − 1 (which is influenced by the HAQ-DI state in *t −* 2) influences the mortality in *t*. *t* = time in years, and *f* = “is a function of”. Note: Each HAQ-DI state should be included in a “triangle” as they all influence the lung and no lung states. However, due to lack of space on the diagram, two triangles were omitted. *dcSSc* diffuse cutaneous systemic sclerosis, *HAQ-DI* Health Assessment Questionnaire-Disability Index

After calibration, a microsimulation structure was constructed, which enabled patients to move between the five HAQ-DI states, to develop major advanced organ involvement, and/or to enter a death state. An illustrative microsimulation was proposed for a patient (simulated over a lifetime horizon) to randomly transition between the five HAQ-DI states, to develop lung/no lung involvement, and to enter a death state at each time cycle. The structure of the model is shown in Fig. 3. In that structure, a female patient starts in a specific HAQ-DI and lung state (defined by baseline characteristics). At the start of each cycle, based on her HAQ-DI state and lung state in the previous cycle, she could evolve towards another HAQ-DI and/or lung state, or die. The equations used to model the transitions in that illustrative model were:

- HAQ-DI state in *t* = *f*(age in *t* − 1, sex, lung status in *t* − 1)
- Lung status in *t* = *f*(HAQ-DI state in *t* − 1, time since disease onset)
- Mortality in *t* = *f*(lung status in *t* − 1, age in *t* − 1, sex) where *t* = time (years); *f* = “is function of”.

Several assumptions were made as these equations were calibrated on data with different time horizons (only lung status was a time-dependent predicted variable). The calibrated equations were:

- HAQ-DI state after 1 year = *f*(age at baseline, sex, lung status at baseline)
- Lung status in year *t* = *f*(HAQ-DI state at baseline, time since disease onset)
- Mortality in *t* = *f*(lung status in *t* − 1, age in *t* − 1, sex)

## Results

### Study population

A total of 4132 dcSSc patients without HAQ-DI score and 690 dcSSc patients with HAQ-DI score assessments were included in the EUSTAR registry. Of the 690 patients, 424 patients (61.4%) had ≥ 2 HAQ-DI score assessments and were included in HAQ-DI score progression and death rate analyses, and 266 patients (38.6%) had only one HAQ-DI score assessment and were included in the death rate analysis (Fig. 1). The proportion of patients with major advanced organ involvement is shown in Table 1. One advanced organ involvement was reported in 14.2% of patients with no HAQ-DI score assessments, in 26.2% with ≥ 1 HAQ-DI score assessment, and in 25.6% with ≥ 2 HAQ-DI score assessments.

The baseline characteristics of patients with ≥ 1 (*N* = 690) and ≥ 2 (*N* = 424) HAQ-DI score assessments are summarized in Table 1. In general, the demographic characteristics of these patients were comparable with those with no HAQ-DI score assessments (*N* = 4132). However, most patients with ≥ 1 (77.5%) and ≥ 2 (77.1%) HAQ-DI score assessments had received immunomodulator treatment, whereas only 32.2% of those with no HAQ-DI data had received treatment (Fig. S1). For patients with ≥ 2 HAQ-DI scores, the mean (standard deviation [SD]) duration between first and second assessment of the HAQ-DI score was 11.8 (9.6) months.

### Predictors of death

Of the 690 patients with ≥ 1 HAQ-DI score assessment, 522 (75.7%) had follow-up information with a median duration of 29.9 months (range 2.1–86.2 months), and 41 (7.9%) patients died. Univariate analysis showed that baseline HAQ-DI score, older age at onset of Raynaud’s phenomenon, corticosteroid treatment > 10 mg/day, and advanced major organ involvement (other than renal involvement) were respectively associated with the risk of death (Table 2). Of the baseline HAQ-DI score, all eight domains were associated with mortality. When testing the eight domains in a multivariable model with stepwise backward selection, only the domains walking and dressing remained as predictors of mortality (Table S3).

**Table 2:** Univariate Cox regression analysis of factors influencing risk of death in patients with ≥ 1 HAQ-DI measurement

Baseline parameter	HR	95% CI for HR	*p* value
Male gender	1.15	0.57, 2.35	0.698
HAQ-DI score	3.01	1.98, 4.60	< 0.001
Age at onset of RP	1.03	1.00, 1.05	0.030
mRSS	1.02	0.99, 1.05	0.303
RNA polymerase III positive	0.04	0.00, 11.11	0.260
Anti-topoisomerase I antibody	0.92	0.45, 1.88	0.817
Immunomodulator treatment	0.86	0.45, 1.63	0.638
Corticosteroids > 10 mg/day	6.29	2.64, 14.98	< 0.001
One advanced organ involvement	3.93	1.99, 7.78	< 0.001
Two advanced organ involvement	8.43	3.40, 20.91	< 0.001
Advanced lung involvement	4.81	2.37, 9.77	< 0.001
Advanced cardiac involvement	6.24	1.47, 26.50	0.013
Advanced GI involvement	2.22	1.06, 4.64	0.035
Advanced PH involvement	6.50	2.66, 15.88	< 0.001
Advanced renal involvement	1.12	0.15, 8.13	0.914

*CI* confidence interval, *GI* gastrointestinal, *HAQ-DI* Health Assessment Questionnaire-Disability Index, *HR* hazard ratio, *mRSS* Modified Rodnan Skin Score, *PH* pulmonary arterial hypertension, *RP* Raynaud’s phenomenon

**Table 3:** Multivariable Cox regression analysis of factors affecting risk of death in patients with ≥ 1 HAQ-DI measurement

Baseline parameter	HR	95% CI for HR	*p* value
HAQ-DI score	2.69	1.71, 4.23	< 0.001
Age at onset of RP	1.02	0.99, 1.04	0.177
mRSS	1.01	0.98, 1.04	0.527
Male gender	1.06	0.48, 2.35	0.888
Immunomodulator treatment	0.41	0.20, 0.84	0.014
Corticosteroids > 10 mg/day	5.41	2.10, 13.97	< 0.001
Advanced organ involvement (reference: no organ involvement)			
One	3.57	1.77, 7.18	< 0.001
Multiple	4.81	1.73, 13.34	0.003

*CI* confidence interval, *HAQ-DI* Health Assessment Questionnaire-Disability Index, *HR* hazard ratio, *mRSS* Modified Rodnan Skin Score, *RP* Raynaud’s phenomenon

Multivariable analysis showed that baseline HAQ-DI score, corticosteroid treatment > 10 mg/day, and major advanced organ involvement (1 or multiple organs) remained predictive of death after adjustment on baseline parameters; a 1-point increase in baseline HAQ-DI score multiplied the risk of death by 2.7 (*p* <  0.001) and multiple advanced major organ involvement multiplied the risk of death by 2.8 (*p* <  0.05) (Table 3).

### Predictors of HAQ-DI score progression

Of patients who had ≥ 2 HAQ-DI score assessments, HAQ-DI score progression after 1 year (± 2 months) was monitored in 62.3% (*n* = 264/424); of those, 78.0% (206/264) also had a HAQ-DI score assessment at 1 year. The mean (SD) change in HAQ-DI score from baseline to 1 year was 0.004 (0.39) for all patients (*n* = 206), 0.02 (0.39) in those with no major advanced organ involvement after 1 year (*n* = 146), − 0.06 (0.38) in those with ≥ 1 major advanced organ involvement after 1 year (*n* = 24/55; 43.6% progressors), and 0.15 (0.27) in those with ≥ 2 major advanced organs involved (*n* = 3/5; 60.0% progressors).

On multivariable analysis (Table 4), patients with high baseline mRSS scores or esophageal symptoms showed worsening function (i.e., increasing HAQ-DI scores) over 1 year, whereas those with higher baseline HAQ-DI scores showed improved functioning (i.e., decreasing HAQ-DI scores). Major advanced organ involvement at baseline was not predictive of HAQ-DI score progression at 1 year.

**Table 4:** Multivariable linear regression analysis of effect of baseline characteristics on HAQ-DI score progression at 1 year

	*B*	95% CI for *B*	*p* value
Constant	− 0.16	− 0.51, 0.20	0.389
HAQ-DI score at baseline	− 0.15	− 0.27, − 0.04	0.009
Age at onset of RP	− 0.002	− 0.01, 0.004	0.518
Male gender	0.02	− 0.18, 0.22	0.834
mRSS	0.01	0.0001, 0.03	0.048
Immunomodulator treatment	0.02	− 0.17, 0.20	0.870
Corticosteroids > 10 mg/day	0.12	− 0.77, 1.01	0.791
Advanced organ involvement^a^	− 0.15	− 0.67, 0.37	0.575
Advanced lung involvement	0.05	− 0.55, 0.65	0.866
Advanced cardiac involvement	0.62	− 0.36, 1.60	0.213
Advanced GI involvement^b^	0.40	− 0.20, 0.99	0.186
Advanced renal involvement	0.47	− 0.56, 1.50	0.367
Esophageal symptoms	0.33	0.14, 0.52	0.001
Stomach symptoms	− 0.04	− 0.25, 0.16	0.670
Intestinal symptoms	0.09	− 0.09, 0.26	0.335

*CI* confidence interval, *GI* gastrointestinal, *HAQ-DI* Health Assessment Questionnaire-Disability Index, *mRSS* modified Rodnan Skin Score, *RP* Raynaud’s phenomenon

^a^Involved organs coded as 0 = none, 1 = one organ involvement, 2 = multiple organ involvement

^b^Malabsorption or ≥ 10% weight loss from baseline

### Transition model for dcSSc

**Fig. 4:**
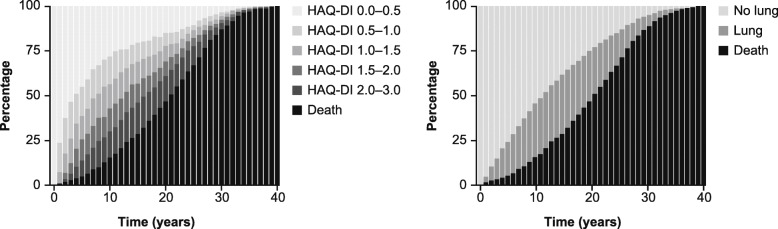
Distribution of dcSSc patients over time between the various HAQ-DI, lung and mortality states as per an illustrative microsimulation that was run over 40 years for a cohort of dcSSc patients. Note: Model was based on 1000 simulated patients with specific baseline characteristics (33% male, age 50 years, HAQ-DI score < 0.5, no lung involvement, and known time since disease onset). *dcSSc* diffuse cutaneous systemic sclerosis, *HAQ-DI* Health Assessment Questionnaire-Disability Index

The illustrative microsimulation model was run over 40 years (with a cycle length equal to 1 year) for a cohort of patients with dcSSc experiencing HAQ-DI states transitions, developing advanced lung involvement, and dying. The distribution of these patients over time between the various HAQ-DI, lung, and mortality states is shown in Fig. 4. This prediction was based on a simulated cohort of 1000 patients with the following baseline characteristics: 33% male; aged 50 years; HAQ-DI score <  0.5; no lung involvement; and time elapsed since disease onset equal to 0. dcSSc patients do not have a linear HAQ-DI score evolution; therefore, a transition-based model enabled a cohort of dcSSc patients to experience both improvement and worsening of HAQ-DI score. By modifying those baseline characteristics and simulating another dcSSc population (e.g., all patients starting in HAQ-DI score > 2.5), one can observe how patients are differently distributed between the various health states along the simulation (e.g., they will experience advanced lung events faster and die sooner).

## Discussion

The objectives of this EUSTAR database analysis were to identify predictors of death (with an emphasis on HAQ-DI score and major advanced organ involvement), to identify predictors of HAQ-DI score progression at 1 year, and to develop a model to predict disease progression in dcSSc using HAQ-DI score, major advanced organ involvement, and death rates. For death outcomes, the study showed that baseline HAQ-DI score, corticosteroid treatment, and advanced major organ involvement (1 or multiple organs) were predictive of death; importantly, the study showed that baseline HAQ-DI score and multiple major advanced organ involvement were equally and independently predictive of death. The link between corticosteroids and mortality risk is difficult to interpret, but they are commonly used for associated arthritis, myositis, or ILD, which all have poor prognosis. Therefore, a bias for indication may apply; corticosteroids > 10 mg/day could be a surrogate indicator for more severe disease with a higher mortality risk.

For HAQ-DI score progression, patients with high baseline mRSS and esophageal symptoms showed worsening HAQ-DI score at 1 year, which may indicate that these measures correlate well in assessing disease progression in patients with dcSSc. Patients with a high baseline HAQ-DI score showed improved functioning, which could be a regression to the mean or may reflect a potential for improvement compared with those who had low HAQ-DI scores at baseline.

A previous study of patients with SSc included in the EUSTAR registry identified multiple predictors of low survival, including male sex, age > 50 years, cardiac impairment (LVEF < 50%), and ILD, although HAQ-DI scores were not reported [20]. In the present study, we can clearly see the importance of the HAQ-DI score as a predictor of death in addition to these predictors (notably major advanced organ involvement); it is also a marker of disease progression that may correlate well with mRSS. This is also consistent with other studies where the HAQ was found to be predictive of mortality in the general population and in patients with rheumatoid arthritis and inflammatory polyarthritis [21–23].

The clinical relevance of the findings from this study is multi-fold. First, this analysis may change physician perception about the HAQ-DI score, and they may now rate it on a comparable level to more objective measures, such as mRSS or organ involvement. Second, the HAQ-DI score could be used as an endpoint in clinical studies of patients with dcSSc. It has been used as an endpoint in recent studies with tocilizumab [8] and abatacept [9] in patients with SSc, and has been shown to be responsive to treatment-induced changes in disease activity. In one study, HAQ-DI score was used separately and as part of the ACR Combined Response Index in Systemic Sclerosis (ACR-CRISS, a composite endpoint that captures cardio-pulmonary-renal involvement and change in mRSS, HAQ-DI score, patient and physician global assessments, and FVC % predicted) [9]; this has been used a primary outcome, and our findings support the value of HAQ-DI score. Third, the HAQ-DI score should be included in health technology assessments of new drug treatments in dcSSc. Many self-evaluations in clinical studies are now mandated by the US Food and Drug Administration [9]. The HAQ-DI score has also been supported in health technology appraisals by the National Institute for Health and Clinical Excellence for oral mycophenolate [24] and rituximab [25] in SSc. Finally, the transition model provides a useful way of monitoring treatment needs over a lifetime horizon, and it could be used in economic models to monitor the impact of new treatments in different patient subgroups.

The strengths of this study include the use of a large, prospective registry and rigorous documentation of dcSSc and advanced organ involvement. In this model, death rates were also calculated using an SMR. This is the first time that SMRs per organ involvement have been calculated, although it was not a key objective of the study. The SMRs were higher than reported in previous studies [5, 26]. In one meta-analysis of 9 studies in 2691 patients with SSc, the pooled SMR was 3.53 (95% CI 3.03, 4.11; *p* <  0.001). In another French study of 625 patients with SSc, overall SMR was 5.73 (95% CI 4.68, 6.94). Taken together, these studies highlight the devastating nature of this condition.

Limitations include the observational design, lack of treatment effect on outcomes, and no inclusion of non-European patients. Regarding the computation of SMRs, we observed a low number of deaths in each HAD-DI category, and this resulted in a non-linear progression of SMRs when increasing the HAQ-DI category. Therefore, this low number of deaths in each of the five HAQ-DI categories may introduce some instability in the results. In addition, the model shown was a simplified illustration of how this tool could work to monitor disease progression. It did not account for any other organ involvement, and only advanced lung involvement was considered a predictor of death (HAQ-DI score is a predictor of death, along with some other baseline characteristics, but these were not considered in order to avoid multicollinearity issues). As SMRs were calculated exclusively in dcSSc patients with non-missing HAQ-DI values or non-missing lung status, it is likely SMRs for dcSSc patients are slightly overestimated. Indeed, it is a known fact that patient in critical condition are more likely to have metabolic and questionnaire-related values recorded. A meta-analysis (nine studies from 1960 to 2010) among SSc patients (not exclusively dcSSc patients) presented an SMR equal to 3.53 (95% CI 3.03, 4.11) [26]. This study also reported a non-significant change of SMR over the past 40 years. Therefore, we deemed the SMRs calculated among dcSSc patients as acceptable.

## Conclusions

This EUSTAR database analysis showed that HAQ-DI scores offer a useful method of monitoring the natural progression of dcSSc in relation to major advanced organ involvement and death. The HAQ-DI score was found to be a significant predictor of mortality risk, thus highlighting the major importance of subjective measures on disease activity and patient well-being. It is hoped that this could evoke a paradigm switch to focusing on more global measures of disease activity in dcSSc, and to rate the HAQ-DI scores in comparison to more objective measures such as major advanced organ involvement. Overall, these findings support the importance of the HAQ-DI score as an endpoint in clinical studies of therapeutic interventions in dcSSc. This analysis also provides a useful model for predicting long-term disease progression in dcSSc.

## Supplementary information

**Additional file 1: Fig. S1.** Immunomodulator treatment received at baseline according to HAQ-DI score assessments. **Table S1.** Results of the multistate model calibration on longitudinal patient-level data (transition intensities between the HAQ-DI states in function of covariates). **Table S2.** Results of a survival model calibration on longitudinal patient-level data (transition intensities between the organ states in function of covariates). **Table S3.** Univariate and multivariable Cox regression analysis of the HAQ-DI domains influencing mortality risk in patients with ≥ 1 HAQ-DI measurement.

## Data Availability

The data that support the findings of this study are available from Professor Yannick Allanore, upon request. Further details on Sanofi’s data sharing criteria, eligible studies, and process for requesting access can be found at: https://www.clinicalstudydatarequest.com.

## Abbreviations

ACR: American College of Rheumatology
CI: Confidence interval
dcSSc: Diffuse cutaneous systemic sclerosis
EULAR: European League Against Rheumatism
EUSTAR: European Scleroderma Trials and Research
GI: Gastrointestinal
HAQ-DI: Health Assessment Questionnaire-Disability Index
HR: Hazard ratio
ILD: Interstitial lung disease
IQR: Interquartile range
LVEF: Left ventricular ejection fraction
mRSS: Modified Rodnan Skin Score
MSM: Multistate modelling
NA: Not available
PH: Pulmonary hypertension
RP: Raynaud’s phenomenon
SD: Standard deviation
SE: Standard error
SMR: Standardized mortality ratio
SSc: Systemic sclerosis
