# Supplementary material for: Health Assessment Questionnaire-Disability Index (HAQ-DI) use in modelling disease progression in diffuse cutaneous systemic sclerosis: an analysis from the EUSTAR database
Source: Arthritis Res Ther. 2020 Oct 28;22:257. doi: 10.1186/s13075-020-02329-2 (PMC7592571; doi:10.1186/s13075-020-02329-2)
Supplement: Supplementary file 1 — Additional file 1: Fig. S1. Immunomodulator treatment received at baseline according to HAQ-DI score assessments. Table S1. Results of the multistate model calibration on longitudinal patient-level data (transition intensities between the HAQ-DI states in function of covariates). Table S2. Results of a survival model calibration on longitudinal patient-level data (transition intensities between the organ states in function of covariates). Table S3. Univariate and multivariable Cox regression analysis of the HAQ-DI domains influencing mortality risk in patients with ≥ 1 HAQ-DI measurement. [file 13075_2020_2329_MOESM1_ESM.docx]

**Additional file 1**

**Fig. S1** Immunomodulator treatment received at baseline according to HAQ-DI score assessments

*HAQ-DI* Health Assessment Questionnaire-Disability Index. *TNF* tumour necrosis factor.

**
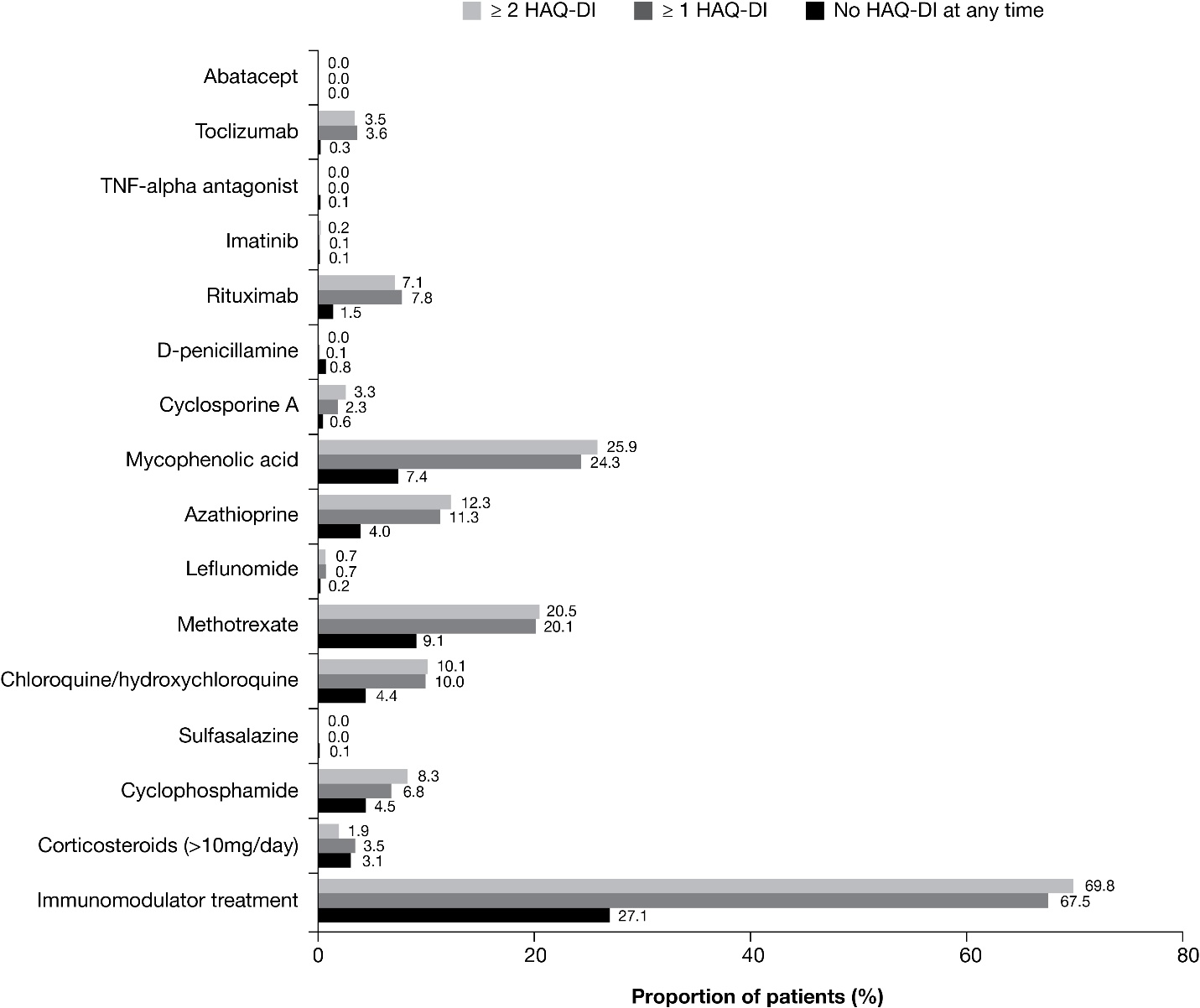
**

**Table S1** Results of the multi-state model calibration on longitudinal patient-level data (transition intensities between the HAQ-DI states in function of covariates)

| **Transition** | **Baseline** | **Age at baseline** | **Sex** | **Lung status at baseline** |
| --- | --- | --- | --- | --- |
| State 1–State 1 | ─0.3046  (─0.4241, ─0.2188) | 0 | 0 | 0 |
| State 1–State 2 | 0.3046  (0.2188, 0.4241) | 1 | 1 | 1 |
| State 2–State 1 | 0.5657  (0.4018, 0.7966) | 1 | 1 | 1 |
| State 2–State 2 | ─1.3265  (─1.7087, ─1.0298) | 0 | 0 | 0 |
| State 2–State 3 | 0.7608  (0.5329, 1.0861) | 1 | 1 | 1 |
| State 3–State 2 | 0.6810  (0.4778, 0.9706) | 1 | 1 | 1 |
| State 3–State 3 | ─1.5477  (─1.9974, ─1.1993) | 0 | 0 | 0 |
| State 3–State 4 | 0.8668  (0.6100, 1.2315) | 1 | 1 | 3.119  (1.190, 8.174) |
| State 4–State 3 | 0.8694  (0.6191, 1.2208) | 1 | 1 | 4.153  (1.656, 10.411) |
| State 4–State 4 | ─1.4340  (─1.8299, ─1.1238) | 0 | 0 | 0 |
| State 4–State 5 | 0.5647  (0.4105, 0.7768) | 1 | 2.346  (1.217, 4.52) | 1 |
| State 5–State 4 | 0.4632  (0.3305, 0.6493) | 0.9726  (0.9501, 0.9957) | 1 | 1 |
| State 5–State 5 | ─0.4632  (─0.6493, ─0.3305) | 0 | 0 | 0 |

*HAQ-DI* Health Assessment Questionnaire-Disability Index.

Note: The intensity represents the instantaneous risk of moving from one state to another state. The intensities form a matrix Q with the diagonal entries defined by a value rendering the row sums equal to zero. The time-specific probabilities of transition P are calculated by taking the exponential of the time-adjusted transition intensities.

$$P\left( t \right)=Exp(tQ)$$

As presented below, the matrix exponential Exp is different from a scalar exponential and we decided not to enter this level of details here. For illustration, let us use a simple example and consider a 2-state HAQ-DI structure. Based on the above intensities, the probability of moving from state 1 to state 2 in one year for a 50-year-old man with a lung involved is given by

$P\left( 1 \right)={1-e}^{-1(0.3046+50+1+1)}$An interested reader can consult <https://cran.r-project.org/web/packages/msm/vignettes/msm-manual.pdf> for more details.

**Table S2** Results of a survival model calibration on longitudinal patient-level data (transition intensities between the organ states in function of covariates)

| **Dependent variables** | **Independent variables and distribution parameters** | **Estimate** | **Lower 95%** | **Upper 95%** | **SE** |
| --- | --- | --- | --- | --- | --- |
| Lung status | meanlog | 2.92014 | 2.13738 | 3.70289 | 0.39937 |
|  | SDlog | 1.50225 | 1.15084 | 1.96096 | 0.20424 |
|  | HAQ-DI state at baseline = [0.5, 1.0] | 0.76557 | ─0.33022 | 1.86136 | 0.55909 |
|  | HAQ-DI state at baseline = [1.0, 1.5] | ─0.00182 | ─0.81985 | 0.8162 | 0.41737 |
|  | HAQ-DI state at baseline = [1.5, 2.0] | ─0.47496 | ─1.23822 | 0.28829 | 0.38942 |
|  | HAQ-DI state at baseline = [2.0, 3.0] | ─0.63921 | ─1.42512 | 0.1467 | 0.40098 |
| PH status | Shape | 1.4702 | 1.3371 | 1.6165 | 0.0712 |
|  | Scale | 30.1265 | 25.5601 | 35.5087 | 2.5266 |
|  | Lung at baseline = Involved | ─0.7333 | ─0.9784 | ─0.4882 | 0.1251 |
| Heart status | Meanlog | 4.642 | 4.229 | 5.055 | 0.211 |
|  | SDlog | 1.595 | 1.402 | 1.815 | 0.105 |
|  | Sex = Male | ─0.736 | ─1.033 | ─0.44 | 0.151 |
|  | Lung at baseline = Involved | ─0.636 | ─1.047 | ─0.224 | 0.21 |
| Kidney status | Meanlog | 6.018 | 5.245 | 6.791 | 0.394 |
|  | SDlog | 2.383 | 2.025 | 2.804 | 0.198 |
| GI status | Meanlog | 2.6551 | 2.4152 | 2.8949 | 0.1224 |
|  | SDlog | 1.5384 | 1.4437 | 1.6394 | 0.0499 |
|  | Lung at baseline = Involved | ─0.7973 | ─1.0411 | ─0.5534 | 0.1244 |
|  | PH at baseline = Involved | ─0.9437 | ─1.4242 | ─0.4632 | 0.2452 |
|  | Age at baseline = [40, 50] years | 0.4117 | 0.1273 | 0.6962 | 0.1451 |
|  | Age at baseline = [50, 60] years | 0.3663 | 0.1003 | 0.6323 | 0.1357 |
|  | Age at baseline = [60, 70] years | 0.5814 | 0.3097 | 0.853 | 0.1386 |
|  | Age at baseline = [70, 80] years | 0.459 | 0.1694 | 0.7485 | 0.1478 |

*GI* gastrointestinal, *HAQ-DI* Health Assessment Questionnaire-Disability Index, *PH* pulmonary arterial hypertension, *SD* standard deviation, *SE* standard error.

**Table S3** Univariate and multivariable cox-regression analysis of the HAQ-DI domains influencing mortality risk in patients with ≥ 1 HAQ-DI measurement

| **Domain** | **HR** | **95% CI for HR** | ***p*-value** |
| --- | --- | --- | --- |
| **Univariate** |  |  |  |
| Walking | 2.58 | 1.84, 3.60 | < 0.001 |
| Common daily activities | 2.30 | 1.64, 3.22 | < 0.001 |
| Arising | 2.17 | 1.58, 2.98 | < 0.001 |
| Dressing | 2.16 | 1.54, 3.02 | < 0.001 |
| Grip | 1.85 | 1.27, 2.69 | < 0.001 |
| Hygiene | 1.83 | 1.38, 2.43 | < 0.001 |
| Eating | 1.72 | 1.27, 2.33 | 0.001 |
| Reach | 1.70 | 1.24, 2.33 | 0.001 |
| **Multivariable** |  |  |  |
| Walking | 2.09 | 1.43, 3.05 | < 0.001 |
| Dressing | 1.58 | 1.08, 2.31 | 0.018 |

*CI* confidence interval, *HAQ-DI* Health Assessment Questionnaire-Disability Index, *HR* hazard ratio.
